# Increased C reactive protein, cardiac troponin I and GLS are associated with myocardial inflammation in patients with non-ischemic heart failure

**DOI:** 10.1038/s41598-021-82592-8

**Published:** 2021-02-04

**Authors:** S. Schwuchow-Thonke, S. Göbel, T. Emrich, V. H. Schmitt, F. Fueting, C. Klank, F. Escher, H. P. Schultheiss, T. Münzel, K. Keller, P. Wenzel

**Affiliations:** 1 Center of Cardiology, Cardiology I, University Medical Center Mainz (Johannes Gutenberg-University Mainz), Langenbeckstr. 1, 55131 Mainz, Germany; 2 Center for Thrombosis and Hemostasis (CTH), University Medical Center Mainz (Johannes Gutenberg-University Mainz), Mainz, Germany; 3 German Center for Cardiovascular Research (DZHK), Partner Site Rhine Main, Mainz, Germany; 4 Department of Diagnostic and Interventional Radiology, University Medical Center Mainz (Johannes Gutenberg University Mainz), Mainz, Germany; 5 Institut Kardiale Diagnostik Und Therapie (IKDT), Moltkestrasse 31, 12203 Berlin, Germany; 6 Departement of Internal Medicine and Cardiology, Charité – Universitätsmedizin Berlin, Campus Virchow Klinikum, Berlin, Germany; 7 German Center for Cardiovascular Research (DZHK), Partner Site, Berlin, Germany

**Keywords:** Biomarkers, Cardiology, Medical research, Signs and symptoms

## Abstract

Inflammatory cardiomyopathy diagnosed by endomyocardial biopsy (EMB) is common in non-ischemic heart failure (HF) and might be associated with adverse outcome. We aimed to identify markers predicting myocardial inflammation in HF. We screened 517 patients with symptomatic non-ischemic HF who underwent EMB; 397 patients (median age 54 [IQR 43/64], 28.7% females) were included in this study. 230 patients were diagnosed with myocardial inflammation, defined as ≥ 7.0 CD3^+^ lymphocytes/mm^2^ and/or ≥ 35.0 Mac1 macrophages/mm^2^ and were compared to 167 inflammation negative patients. Patients with myocardial inflammation were more often smokers (52.4% vs. 39.8%, *p* = 0.013) and had higher C-reactive protein (CRP) levels (5.4 mg/dl vs. 3.7 mg/dl, *p* = 0.003). In logistic regression models CRP ≥ 8.15 mg/dl (OR 1.985 [95%CI 1.160–3.397]; *p* = 0.012) and Troponin I (TnI) ≥ 136.5 pg/ml (OR 3.011 [1.215–7.464]; *p* = 0.017) were independently associated with myocardial inflammation, whereas no association was found for elevated brain natriuretic peptide (OR 1.811 [0.873–3.757]; *p* = 0.111). In prognostic performance calculation the highest positive predictive value (90%) was detected for the combination of Global longitudinal strain (GLS) ≥ -13.95% and TnI ≥ 136.5 pg/ml (0.90 (0.74–0.96)). Elevated CRP, TnI and GLS in combination with TnI can be useful to detect myocardial inflammation. Smoking seems to predispose for myocardial inflammation.

## Introduction

Heart failure (HF) is a global health problem which affects approximately over 37 million people world wide^1^. According to data from the Framingham Heart Study, the lifetime risk of developing HF is estimated to be 20% for the ages between 40 and 80 years^2^. It is caused by structural, but also functional cardiac abnormalities, which result in loss of myocardial function^3^. However, in the absence of coronary artery disease, which is the leading cause of HF^4–6^, inflammatory cardiomyopathy is common, particularly in HF with reduced ejection fraction (HFrEF). Thereby, according to the report of the 1995 World Health Organization/International Society and Federation of Cardiology Task Force on the Definition and Classification of Cardiomyopathies, inflammatory cardiomyopathy is defined as cardiac dysfunction in co-prevalence with inflammatory disease of the myocardium established by immunological, histological and immunohistochemical criteria^7^. The exact incidence of inflammatory cardiomyopathy underlying HF is not known. However, according to postmortem analysis, inflammatory cardiomyopathy or myocarditis seems to account for approximately 40% of the sudden cardiac deaths in the young^8^. Regarding endomyocardial biopsy (EMB) studies myocardial inflammation can be diagnosed in 9 to 23% of the patients with non-ischemic cardiomyopathy^9,10^. According to the above mentioned definitions, inflammatory cardiomyopathy can only be diagnosed on basis of EMB results^11,12^. Although the results of studies addressing the immunosuppressive therapy of inflammatory cardiomyopathy remain controversial^13–15^, results of the TIMIC trial suggest that immunosuppressive therapy in patients with virus negative inflammatory cardiomyopathy may be an effective and safe option for recovery of cardiac function in addition to optimal medical therapy^16^. Inflammatory cardiomyopathy can progress rapidly and may require immediate immunosuppressive therapy to prevent adverse outcomes. Thus, we aimed to identify predictors of myocardial inflammation in HF patients in order to improve diagnostic and therapeutic management.

## Methods

### Study population

**Figure 1:**
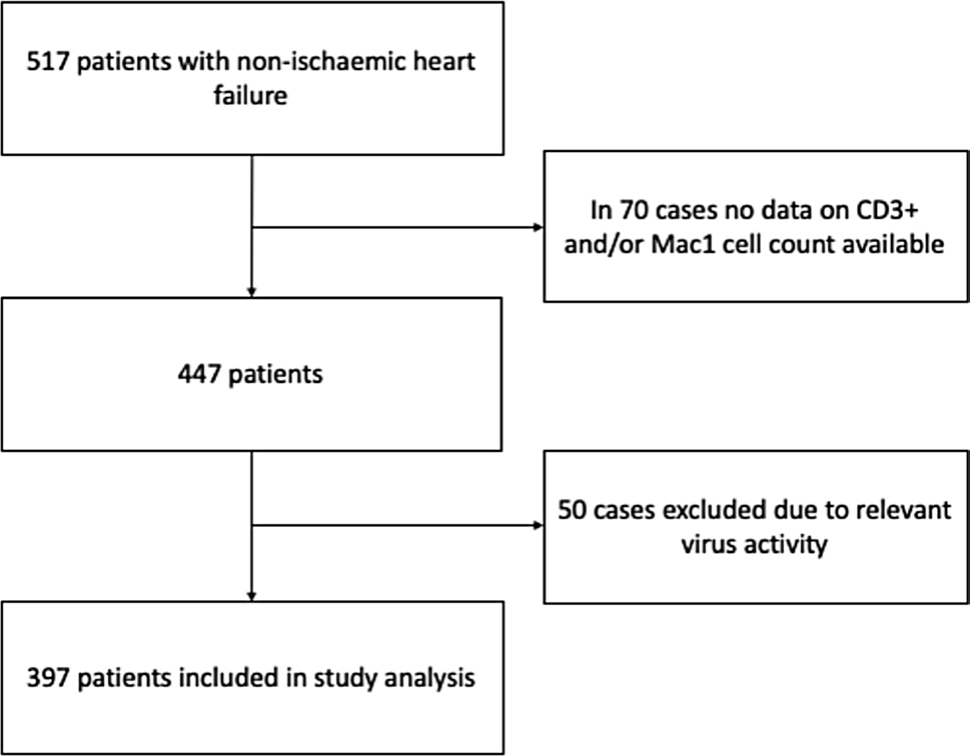
Study flow chart.

Patients presenting with symptoms of HF at the Heart Failure Outpatient clinic, the emergency department or chest pain unit of the University Medical Centre Mainz between 14/10/2012 and 18/12/2018, who underwent EMB were enrolled in this retrospective monocentric analysis. Decision to obtain EMB of the patients was based on guidelines published of the AHA, the ACC and the ESC in 2007^17^. Ischemic HF, valvular HF and systemic disease with known cardiac involvement were ruled out prior to EMB. The analysis of the patients’ medical records involved the following data: personal medical history, clinical presentation, laboratory values, echocardiography and the finding of the EMB, including viral activity. The exclusion criteria are presented in Fig. 1 and comprise missing CD3+ and/or Mac1 cell counts or presence of relevant virus activity. All data were obtained from individuals enrolled between 2013 and 2018 in the retrospective monocentric Mainz Endomyocardial Biopsy in Heart Failure Study (My Biopsy-HF Study, DRKS #22178), which was approved by the Ethics Committee of Rhineland Palatinate to be in accordance with the legal regulations and the declaration of Helsinki. Informed consent was obtained from all included individuals to use EMB tissue samples for further scientific purpose.

### Echocardiography

Transthoracic echocardiography was performed in our echocardiography lab by trained and certified specialists of our department using iE33 Philips, a General Electrics E9 or Siemens Acuson s2000 machines. All examinations were performed with the patient in the standard left lateral position while apnea or quiet breathing. Routinely 2–4 cardiac cycles were obtained at frame rates of 50–100 fps and digitally transferred into a picture archiving and communication system (Xcelera) for offline analysis. Left ventricular (LV) ejection fraction (LVEF) was assessed by biplane Simpson’s method in the apical four and two chamber view. For the assessment of cardiac structure, LV internal diameters were measured. LVEF was categorized into classes LVEF ≥ 50% (HF with preserved LVEF = HFpEF), LVEF 40–49% (HF with mid-range reduced LVEF = HFmrEF) and LVEF < 40% (HFrEF)^3^. Global longitudinal strain (GLS) was measured offline utilizing QLab 13.0 (Philips Healthcare, Hamburg, Germany) automatic speckle-tracking software. The tracking result was visually controlled and manually readjusted if necessary. In case of persistent poor tracking quality, datasets were excluded from analysis. GLS was automatically calculated by average of GLS in apical four, three and two chamber view.

### Endomyocardial biopsy and myocardial inflammation

EMB was performed using a biopsy forceps (Medwork bioptom, 180 cm, 1.8 mm, Cat.-No. BIO-C4-18-180) by taking up to eight biopsies either from the right ventricular septum or from the lateral wall of the LV. In case of LV biopsy the procedure was carried out via the right or left femoral artery or via trans-radial cardiac catheterization as reported before^18^. In case of right heart biopsy, the femoral vein was used. Immediately after sampling the biopsy, the specimens were stabilized in a solution to preserve ribonucleic acid (RNA) integrity (RNAlater, Ambion Inc., Austin, Texas) and were sent for further examination to a specialized laboratory approved by Food and Drug Administration [Institut Kardiale Diagnostik und Therapie (IKDT), Berlin, Germany]. For immunohistological evaluation, heart muscle tissue probes fixated in RNA*later* were embedded in Tissue Tec (SLEE, Mainz, Germany) and immediately snap‐frozen in methyl butane which had been cooled in liquid nitrogen, and then stored at – 80 °C until processing. Embedded specimens were cut serially into cryosections of 5 mm thickness and placed on 10% poly‐l‐lysine‐pre‐coated slides. As part of a routinely performed work up in case of unclear heart failure histological and immunohistochemical examination as well as virus detection was conducted. Using specific antibodies inflammatory processes were detected by identifying immune cell infiltration and expression of cell adhesion molecules. The specimens of the patients in this study were tested for CD3-positive lymphocytes (Dako; dilution 1:25), CD11a^+^/LFA-1^+^ lymphocytes (ImmunoTools; dilution 1:250), macrophages Mac-1 macrophages (ImmunoTools; dilution 1:500), Perforin positive (cytotoxic T Cells) (clone dG9, BD Bioscience; dilution 1:150) and the expression of adhesion molecules HLA-1 (Dako; dilution 1:2000) and ICAM-1 (ImmunoTools; dilution 1:800). Myocardial inflammation was defined as ≥ 7.0 CD3^+^ lymphocytes/mm^2^ and/or ≥ 35.0 Mac1 macrophages/mm^2^ in accordance with previous published studies^19^. Diagnosis of relevant myocardial virus activity was performed by polymerase chain reaction (PCR) detecting the genomic sequences of viruses that most commonly cause myocarditis. This included: enterovirus, adenovirus, human cytomegalovirus, herpes simplex virus, Epstein–Barr virus, human herpesvirus 6, parvovirus B19 and influenza A and B viruses. Virus load was calculated via quantitative PCR methods. In case of relevant myocardial virus activity patients were excluded from analysis.

### Statistical analysis

Descriptive statistics for relevant baseline comparisons of symptomatic non-valvular, non-ischemic heart failure patients, who underwent EMB, stratified according inflammation (≥ 7.0 CD3+ lymphocytes/mm^2^ and/or ≥ 35.0 Mac1 macrophages/mm^2^) are provided as median and interquartile range (IQR) or absolute numbers and corresponding percentages. We tested the continuous variables of the groups (inflammation vs. no inflammation) using the Mann–Whitney-U test and categorical variables with the Fisher’s exact or the chi^2^ test, as appropriate.

Receiver operating characteristics (ROC) curves were calculated for TnI, BNP, CRP and GLS with regard to myocardial inflammation and the area under the curve (AUC) is presented with the corresponding 95% CI. Additionally, patient cohort-optimised cut-off values of these mentioned laboratory markers with regard to myocardial inflammation were calculated based on ROC analyses using Youden index quantification. The software SPSS (version 23.0; SPSS Inc., Chicago, Illinois) was used for computerized analysis. *p* values of < 0.05 (two-sided) were considered to be statistically significant.

Univariate and multivariate logistic regression models were analyzed to investigate predictors of myocardial inflammation in heart failure patients, thereby using patient-optimised cut-off values derived from the ROC analyses. Results are presented as odds ratio (OR) and 95%CI. The multivariate regression models were adjusted for (1) age and sex, obesity and (2) cardiovascular risk factors (CVRF) including history of smoking, arterial hypertension, diabetes mellitus and hyperlipoproteinaemia.

## Results

Overall, 517 patients with symptomatic non-valvular, non-ischemic HF who underwent EMB between 2012 and 2018 were screened. Results from EMB with CD3+ and/or Mac1 cell count were available in 447 cases. 50 cases were excluded due to relevant virus activity (Fig. 1). Thus, 397 patients remained in this study and were analysed. Patient characteristics are displayed in Table 1. In brief, median age was 54 years [IQR 43/64] and 71.3% of the patients were males. Median left ventricular ejection fraction (LVEF) was calculated with 30.0% [20.0/40.0] and median left ventricular enddiastolic diameter (LVEDD) with 5.9 mm [5.2/6.6] accompanied by an elevated median BNP value of 447 pg/ml [137.0/1188.5]. GLS was reduced with − 8.8% [− 12.25/− 6.0]. Regarding symptoms, 71.7% patients were admitted with dyspnea (43.7% NYHA functional class III or IV), 32.4% complained about angina pectoris at time of admission and in 19.1% edema were detectable. The duration of symptoms differed: In 36.4% of the patients symptoms started not longer than 2 weeks, 30.3% had symptoms between 2 weeks and 3 months and 32.6% suffered from symptoms more than 3 months duration before admission. In immunohistochemistry, median counts of CD3^+^ and Mac1 were 6.470/mm^2^ [IQR 2.0/16.085] and 33.6/mm^2^ [16.9/58.1), respectively, defining a cut-off of ≥ 7.0 CD3^+^ lymphocytes/mm^2^ and/or 35.0 Mac1 macrophages/mm^2^.

**Table 1:** Baseline characteristics.

N	397
Age in years	54 [43/64]
Male	283 [71.3%, n = 397]
BMI in kg/m^2^	26.8 [24.0/30.0]
**Comorbidities and risk factors**	
Diabetes mellitus	64 [16.1%, n = 397]
Hypertension	193 [48.6%, n = 397]
Alcohol	26 [6.6%, n = 394]
History of smoking	186 [47.1%, n = 395]
Hyperlipoproteinaemia	66 [16.7%; n = 396]
**Symptoms at admission**	
NYHA I	88 [28.3%, n = 311]
NYHA II	87 [28.0%, n = 311]
NYHA III	83 [26.7%, n = 311]
NYHA IV	53 [17.0%, n = 311]
Angina pectoris	128 [32.4%; n = 395]
Cough	38 [9.6%; n = 395]
History of VTs	29 [7.3%; n = 392]
Common cold	86 [23.3%; n = 369]
Nausea	45 [11.4%, n = 395]
Oedema	76 [19.1%; n = 390]
Palpitation	55 [13.9%; n = 395]
Syncope	19 [4.8%; n = 395]
**Time since onset**	
≤ 2 weeks	142 [36.4%; n = 390]
> 2 weeks, ≤ 3 months	118 [30.3%; n = 390}
> 3 months	127 [32.6%; n = 390]
**Echocardiographic and parameters**	
LVEF in %	30.0 [20.0/40.0]
GLS in %	-8.8 [-12.25/-6.0]
LVEDD in mm	5.9 [5.2/6.6]
LVEDP in mmHg	18 [12/26]
**Laboratory measures**	
BNP in pg/ml	447 [137/1188.5]
CRP in mg/dl	4.5 [1.7/15.0]
TnI in pg/ml	24.9 [9.2/71.5]
Hb in g/dl	14.3 [13.1/15.4]
**Immunhistochemistry**	
CD3^+^ Cells/mm^2^	6.470 [2.0/16.085]
Mac-1 Cells/mm^2^	33.6 [16.9/58.1]
CD11^+^ Cells/mm^2^	16.540 [8.8/32.105]
Perforin positive Cells/mm^2^	2.4 [1.01/5.2]
HLA1 in % AF	6.63 [5/8.70]
ICAM-1 Cells/mm^2^	2.4 [1.57/3.2]

Absolute and relative frequencies of echocardiographic markers, cardiovascular risk factors and comorbiditis, symptoms at admission and laboratory parameters. BMI = Body Mass Index kg/m^2^; LVEF = left ventricular ejection fraction in %; LVEDD = left ventricular enddiastolic diameter in mm; LVEP = left ventricular enddiastolic pressure in mmHg; NYHA = New York Heart Associaton; VTs = ventricular tachykardia; BNP = Brain natriuetic peptide in pg/ml; CRP = C-reactive protein in mg/l; Hb = Haemoglobin in g/dl; TnI = Troponin I in pg/ml; AF = Area fraction

### Comparison of symptomatic non-valvular, non-ischemic heart failure patients, who underwent EMB, stratified for presence of myocardial inflammation

**Figure 2:**
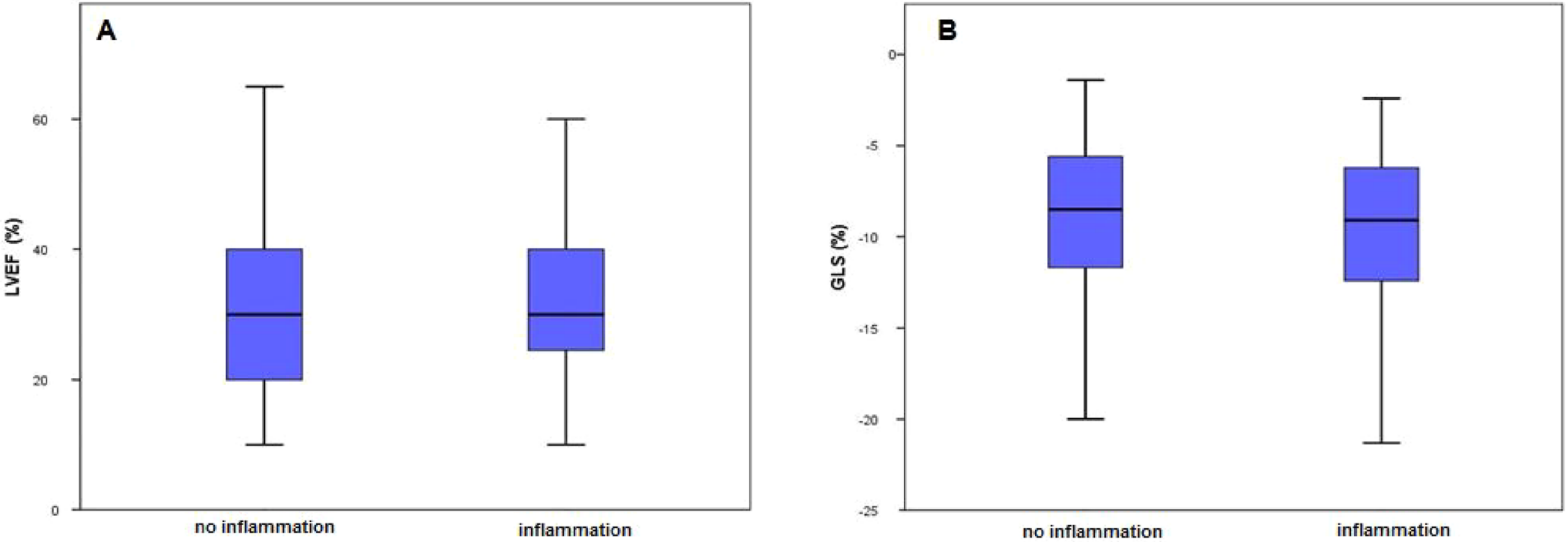
Boxplots showing 1st quartile, median and 3rd quartile values of: (**A**) LVEF = left ventricular ejection fraction in % (not significant); (**B**) GLS = global longitudinal strain in % (not significant);

**Figure 3:**
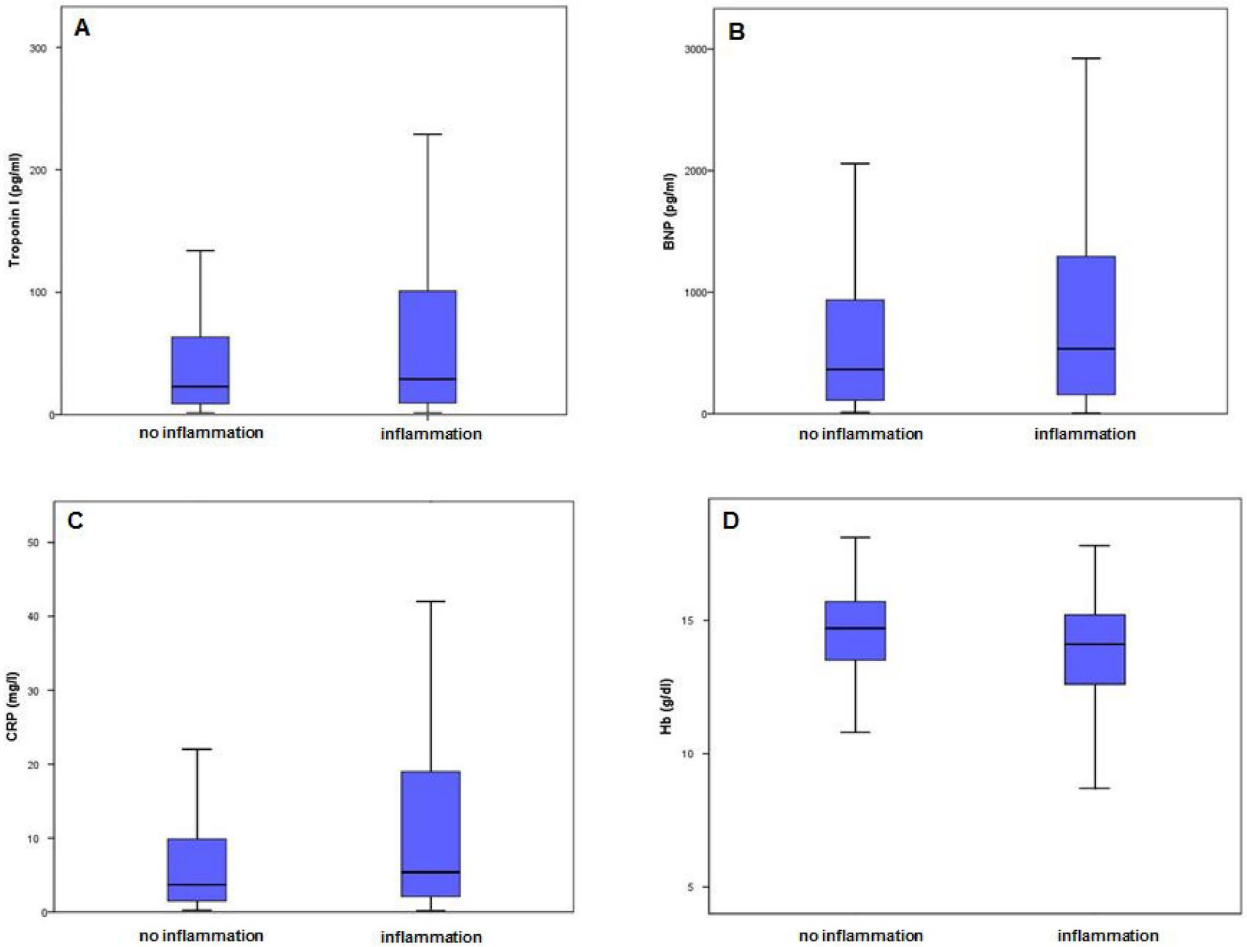
Boxplots showing 1st quartile, median and 3rd quartile values of: (**A**) Troponin I in pg/ml (not significant); (**B**) Brain natriuretic peptide (BNP) in pg/ml (not significant); (**C**) C-reactive protein (CRP) in mg/l (*p* = 0.003); (**D**) Hemoglobin (Hb) in g/dl (*p* = 0.001).

In 230 patients (57.9%), myocardial inflammation was diagnosed. Patients with and without inflammatory cardiomyopathy were of comparable age and revealed no differences regarding presented symptoms at admission. History of smoking (52.4% vs. 39.8%, *p* = 0.013) was more prevalent in HF patients with myocardial inflammation. While the groups did not differ regarding mean values of LVEF, GLS (Table 2, Fig. 2) and mean values of TnI and BNP (Table 2, Fig. 3), mean values of CRP (5.4 [2.1/19] vs. 3.7 [1.5/10], *p* = 0.003) were higher and median hemoglobin levels (14.1 [12.6/15.2] vs. 14.7 (13.5/15.7] g/dl, *p* = 0.001) were lower in patients with myocardial inflammation (Table 2, Fig. 3).

**Table 2:** Comparison of patients with inflammation and no inflammation.

N	Inflammation (230)		No Inflammation (167)		*p* value
Age in years	54 [43/65]		54 [43/62]		0.795
Male	156 [67.8%; n = 230]		127 [76%; n = 167]		0.074
BMI in kg/m^2^	26.0 [23.0/30.0]		27.0 [24.0/30.70]		0.239
**Comorbidities and risk factors**					
Diabetes mellitus	41 (17.8%; n = 230)		23 (13.8%; n = 167)		0.278
Hypertension	110 (47.8%; n = 230)		83 (49.7%; n = 167)		0.712
Alcohol	18 (7.9%; n = 228)		8 (4.8%; n = 166)		0.225
History of smoking	120 (52.4%; n = 229)		66 (39.8%; n = 166)		**0.013**
Hyperlipoproteinaemia	41 (17.8%; n = 230)		25 (15.1%; n = 166)		0.466
**Symptoms at admission**					
NYHA > 2	79 (43.9%, n = 180)		57 (43.5%; n = 131)		0.947
Angina pectoris	73 (31.9%; n = 229)		55 (33.1%; n = 166)		0.793
Cough	25 (10.9%; n = 229)		13 (7.8%; n = 166)		0.305
History of VTs	19 (8.4%; n = 227		10 (6.1%; n = 165)		0.388
Common cold	51 (23.6%; n = 216)		35 (22.9%; n = 153)		0.869
Nausea	26 (11.4%; n = 229)		19 (11.4%; n = 166)		0.977
Oedema	46 (20.4%; n = 226)		30 (18.3%; n = 164)		0.612
Palpitations	30 (13.1%; n = 229)		25 (15.1%; n = 166)		0.579
Syncope	10 (4.4%; n = 229)		9 (5.4%; n = 166)		0.629
**Echocardiographic measures**					
LVEF in %	30.0 [24.25/40.0]		30.0 [20.0/40.0]		0.712
GLS in %	-9.1 [-12.4/-6.15]		-8.5 [-11.8/-5.6]		0.239
LVEDD in mm	5.9 [5.025/6.575]		6.0 [5.3/6.625]		0.126
LVEDP in mmHg	19 [13/28]		17 [11.25/24.75]		0.230
**Laboratory measures**					
BNP pg/ml	534.0 [155.0/1297.0]		364.5 [111.0/941.5]		0.116
TnI in pg/ml	29.0 [9.425/102.750]		22.8 [8.75/64.35]		0.265
CRP in mg/l	5.4 [2.1/19.0]		3.7 [1.5/10.0]		**0.003**
Hb in g/dl	14.1 [12.6/15.2]		14.7 [13.5/15.7]		**0.001**

Absolute and relative frequencies of echocardiographic markers, cardiovascular risk factors and comorbiditis, symptoms at admission and laboratory parameters. BMI = Body Mass Index kg/m^2^; LVEF = left ventricular ejection fraction in %; GLS = global longitudinal strain in %; LVEDD = left ventricular enddiastolic diameter in mm; LVEDP = left ventricular enddiastolic pressure in mmHg; NYHA = New York Heart Associaton; VTs = ventricular tachykardia; BNP = Brain natriuretic peptide in pg/ml; CRP = C-reactive protein in mg/l; Hb = Hemoglobin in g/dl; TnI = Troponin I in pg/ml. *P*-values are bold if they are below the significance level cut-off of 0.05.

### Predictors of myocardial inflammation

ROC curves demonstrated only a moderate prognostic performance of CRP for prediction of myocardial inflammation with acceptable specificity and positive predictive values (Table 3, supplementary Figure 1). The computed best cut-off (according Youden-Index calculation) for CRP was 8.15 mg/l. In contrast, TnI, BNP and GLS were accompanied by a low prognostic performance to predict myocardial inflammation shown in the ROC curves. The computed best cut-off (according Youden-Index calculation) for TnI was 136.5 pg/ml, for BNP 1030 pg/ml and for GLS -13.95% (Table 3). Nevertheless, when calculating best cut-offs (according Youden-Index calculation), the cut-offs were able to differentiate between myocardial inflammation vs. no cardiac inflammation.

**Table 3:** Prognostic performance of TnI, BNP, CRP and GLS for prediction of myocardial inflammation.

Parameter	Best cut-off (Youden index)	AUC (95% CI)	*p* value	Sensitivity (95% CI)	Specificity (95% CI)	PPV (95% CI)	NPV (95% CI)
TnI	136.5 pg/ml	0.54 (0.47–0.60)	0.267	0.21 (0.16–0.27)	0.91 (0.85–0.95)	0.78 (0.66–0.87)	0.43 (0.37–0.48)
BNP	1030.5 pg/ml	0.56 (0.49–0.63)	0.116	0.34 (0.27–0.42)	0.79 (0.71–0.86)	0.70 (0.59–0.79)	0.45 (0.38–0.52)
CRP	8.15 mg/l	0.59 (0.53–0.65)	**0.003**	0.43 (0.37–0.50)	0.71 (0.64–0.78)	0.68 (0.60–0.75)	0.47 (0.41–0.53)
GLS	− 13.95%	0.46 (0.38–0.53)	0.239	0.88 (0.81–0.92)	0.14 (0.08–0.22)	0.58 (0.51–0.64)	0.45 (0.29–0.62)

*P*-values are bold if they are below the significance level cut-off of 0.05.

**Figure 4:**
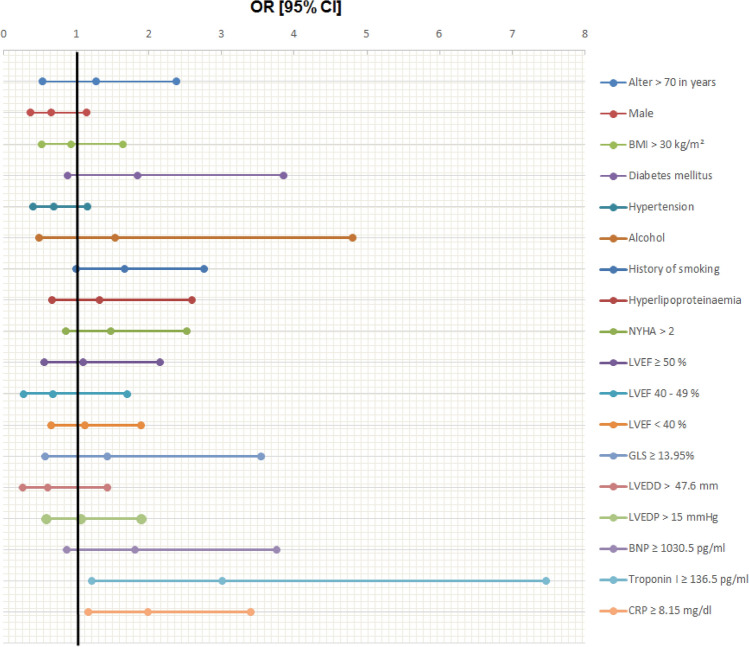
Predictors for inflammation (Forest plot). OR (Odds Ratio) and 95% CI (Confidence Intervall) displayed in a Forest plot after adjustment for age/sex/CVRF. Absolute values are shown in Table 4**.**

In multivariate logistic regression analyses after adjustment for CVRF, elevated TnI values (≥ 136.5 pg/ml) (OR 3.011 [95%CI 1.215–7.464]; *p* = 0.017) as well as elevated CRP values (≥ 8.15 mg/l) (OR 1.985 [95%CI 1.160–3.397], *p* = 0.012) were independently associated with myocardial inflammation. No association was detected for elevated BNP (≥ 1030.5 pg/ml) values (OR 1.811 [95%CI 0.873–3.757]; *p* = 0.111). Regarding echocardiographic parameters, LVEF categories and reduced GLS (≥ − 13.95%) were not associated with myocardial inflammation (Table 4, Fig. 4). However when combining laboratory parameters with echocardiographic parameters, a strong association with myocardial inflammation was found for the combined variable consisting of reduced GLS and elevated TnI (OR 9.633 [95%CI 2.027–45.769]; *p* = 0.004). Additionally, but to a smaller extent, the combination of TnI and CRP was also independently associated with myocardial inflammation (OR 5.761 [95% 1.240–26.771], *p* = 0.025) (Supplementary table 1). Furthermore, supplementary table 2 illustrates the prognostic performance of combined parameters to predict myocardial inflammation. While all parameter-combinations had only moderate sensitivity (21–39%), the specificity was better: 0.71 (0.62–0.79) for GLS and elevated CRP, 0.95 (0.91–0.98) for positive TnI and elevated CRP and 0.96 (0.90–0.99) for the combination of GLS and TnI. Remarkably, highest positive predictive value was also detected for the combination of GLS and TnI 0.90 (0.74–0.96). The negative predictive values were only moderate in all of these combinations (41–46%).

**Table 4:** Predictors of inflammation.

	Adjustment for					
	crude		+ Age/Sex/Obesity		+ Age/Sex/Obesity/CVRF	
	OR [95% CI]	*p* value	OR [95% CI]	*p* value	OR [95% CI]	*p* value
Age > 70 in years	1.2 [0.683–2.108]	0.525	1.042 [0.522–2.080]	0.907	1.127 [0.533–2.382]	0.754
Male	0.664 [0.423–1.042]	0.075	0.701 [0.408–1.205]	0.199	0.652 [0.373–1.140]	1.333
BMI > 30 kg/m^2^	0.943 [0.553–1.605]	0.828	0.940 [0.547–1.615]	0.823	0.930 [0.528–1.638]	0.802
**Comorbidities and risk factors**						
Diabetes	1.358 [0.780–2.365]	0.279	1.709 [0.833–3.506]	0.144	1.843 [0.882–3.848]	0.104
Hypertension	0.928 [0.623–1.282]	0.712	0.763 [0.458–1.272]	0.300	0.685 [0.405–1.158]	0.157
Alcohol	1.693 [0.718–3.993]	0.229	1.353 [0.479–3.818]	0.568	1.532 [0.488–4.804]	0.465
History of Smoking	1.668 [1.113–2.500]	**0.013**	1.537 [0.938–2.520]	0.088	1.659 [1.000–2.753]	0.050
Hyperlipoproteinaemia	1.223 [0.711–2.106]	0.467	1.241 [0.646–2.386]	0.517	1.315 [0.667–2.590]	0.429
NYHA > 2	1.015 [0.645–1.599]	0.947	1.364 [0.811–2.294]	0.242	1.469 [0.857–2.518]	0.162
**Echocardiographic measures**						
LVEF ≥ 50%	0.966 [0.545–1.711]	0.906	1.175 [0.604–2.285]	0.635	1.092 [0.555–2.149]	0.798
LVEF 40–49%	0.957 [0.466–1.963]	0.904	0.703 [0.302–1.638]	0.415	0.678 [0.271–1.700]	0.408
LVEF < 40%	1.081 [0.702–1.664]	0.724	1.077 [0.645–1.800]	0.776	1.116 [0.659–1.888]	0.683
GLS ≥ -13.95%	1.126 [0.527–2.407]	0.759	1.396 [0.569–3.423]	0.466	1.425 [0.573–3.542]	0.446
LVEDD > 47.6 mm	0.455 [0.222–0.934]	**0.032**	0.570 [0.246–1.317]	0.188	0.611 [0.262–1.424]	0.253
LVEDP > 15 mmHg	1.075 [0.681–1.696]	0.756	1.072 [0.608–1.888]	0.811	1.056 [0.588–1.895]	0.856
**Laboratory measures**						
BNP ≥ 1030.5 pg/ml	1.928 [1.084–3.428]	**0.025**	1.837 [0.904–3.735]	0.093	1.811 [0.873–3.757]	0.111
Troponin I ≥ 136.5 pg/ml	2.660 [1.345–5.260]	**0.005**	3.121 [1.291–7.547]	**0.012**	3.011 [1.215–7.464]	**0.017**
CRP ≥ 8.15 mg/l	1.899 [1.238–2.911]	**0.003**	2.009 [1.191–3.386]	**0.009**	1.985 [1.160–3.397]	**0.012**

OR (Odds Ratio), 95% CI (Confidence intervalls) and *p* values in a crude and multivariate logistic regression analysis. BMI = Body Mass Index kg/m^2^; LVEF = left ventricular ejection fraction in %; GLS = global longitudinal strain in %; LVEDD = left ventricular enddiastolic diameter in mm; LVEDP = left ventricular enddiastolic pressure in mmHg; NYHA = New York Heart Associaton; BNP = Brain natriuetic peptide in pg/ml; CRP = C-reactive protein in mg/l. *P*-values are bold if they are below the significance level cut-off of 0.05.

Concerning the interrelation of cardiovascular risk factors with myocardial inflammation, only smoking was associated with myocardial inflammation in crude logistic regression analysis (OR 1.6687 [95%CI 1.113–2.500]; *p* = 0.013), but this association showed only a trend towards significance in the fully adjusted model (OR 1.659 [95%CI 1.000–2.753]; *p* = 0.050) (Table 4, Fig. 4).

## Discussion

The key findings of the present study are that (I) elevated CRP and TnI levels are associated with inflammatory cardiomyopathy in patients with non-ischemic, non valvular heart failure; (II) TnI levels ≥ 136.5 pg/ml and CRP levels ≥ 8.15 mg/l are potential predictors of myocardial inflammation; (III) common heart failure biomarkers such as BNP and reduced LVEF were not predictive for myocardial inflammation and; (IV) GLS might add a benefit to predict myocardial inflammation when combined with TnI.

A growing body of evidence indicates that myocardial inflammation may lead to severe cardiac damage, deteriorate into dilated cardiomyopathy and be accompanied by poor prognosis. This implies that early diagnosis using predictive markers may avert poor outcome^20^, since immunosuppressive therapy can significantly improve the prognosis of HF patients with presence of myocardial inflammation^16^. Thus, readily available markers like the ones identified in our study might help to select patients for EMB and for shorter control follow-up examinations. Although cardiac MRI diagnostics has become increasingly important in the diagnosis of myocardial inflammation^21,22^, sensitivity and specificity of MRI findings are not unequivocal. Since EMB can be performed easily as part of coronary catheter examination^18^ and should be carried out as early as possible if there is clinical or laboratory suspicion of inflammatory myocardial disease, our study results might help to accelerate the diagnostic work-up of HF.

The association of elevated CRP and TnI with myocardial inflammation was independent of age and sex, supporting earlier findings by Lauer et al., who reported that patients with elevated troponin T values were more likely to have EMB-proven myocarditis^23^. Because cardiac troponins are detectable in the blood for approximately one week^24^ and have a half-life of 2h^25^, elevated troponins are suitable to indicate permanent myocardial cell injury^26,27^, which can be explained by inflammatory infiltration with consecutive cell damage documented in the EMB. Contrary to our results, Sramko et al. demonstrated that troponin was not able to distinguish between patients with idiopathic dilated cardiomyopathy or inflammatory dilated cardiomyopathy arguing that troponin indicates myocardial injury without indicating weather its due to myocardial inflammation or simply caused by myocardial wallstress related to heart failure^28^. However, the study sample size was small, and EMB was only performed on the right ventricle making a sampling error more likely.

Similar to positive troponin values, positive CRP values were associated with detection of inflammation in EMB. These results go in line with observation by Liu et al., who demonstrated that highly sensitive CRP levels were higher in patients with EMB-confirmed myocarditis than in patients without myocarditis^29^. However, it has to be pointed out that the sensitivity for highly sensitive CRP in detecting myocarditis was only 50.1% whereas the specificity was 80.7%. Since CRP is also increased in acute myocardial infarction^30^ or in rheumatic valve disease^31^. CRP should not be interpreted alone, but in conjunction with cardiac troponin. Moreover, as the present study indicates, cut-off values for TnI and CRP seem to be higher with regard to myocardial inflammation compared to cut-off values used for diagnosis of acute coronary syndrom. Further studies are needed to prove those cut-off values in patients with myocardial inflammation.

Interestingly GLS was not able to distinguish between EMB proven myocardial inflammation and no inflammation. Escher et al. demonstrated before, that patients with acute myocarditis had reduced GLS values. After a follow-up period of 6.2 months patients with persistent inflammation had worse GLS values than patients without inflammation^32^. However in contrast to our study GLS values were compared when LVEF had already been improved. Eventhough it has been shown that GLS is a superior predictor of adverse cardiac events compared with LVEF^33,34^, studies demonstrate that in general and irrespectively of outcome prediction, GLS correlates well with LVEF in heart failure patients^35^. Therefore the results of our study suggest that GLS alone is not able to identify patients with myocardial inflammation in the presence of reduced LVEF. Nevertheless, the combined evaluation of GLS and Troponin values were the second strongest predictor of myocardial inflammation in multivariate regression analysis. Additionally, the best prognostic performance was found for the combination of GLS and Troponin. In particular, the high positive prognostic value of 90% to predict myocardial inflammation is promising. Thus, the combination of GLS and Troponin may help to identify patients with myocardial inflammation in the future and could add a benefit in the diagnosis and follow up treatment of those patients.

Interestingly, smoking was associated with myocardial inflammation in our study. In a very small study sample, it was shown, that in patients hospitalized for acute myocarditis smoking was the most prevalent cardiovascular risk factor^36^. Recently, Detorakis et al. reported a significant correlation of smoking habits with late gadolinium enhancement extent in cardiac MRI in patients with clinically suspected myocarditis^37^. Prolonged exposure to tobacco smoke may cause cardiovascular cell damage, suggesting that increased myocardial cell necrosis and cell death would make myocardial inflammatory infiltration more likely^38^.

Another finding of the present study was that patients with myocardial inflammation had lower hemoglobin levels. It is well known that anemia of inflammation or anemia of chronic disease is primarily a disorder of iron distribution^39^ and even low grade inflammation is associated with lower hemoglobin levels^40^. As experimental models have demonstrated that low cardiac iron levels promote heart failure^41,42^ the link between the observed lower hemoglobin levels could be an effect of possibly local iron distribution disorders due to myocardial inflammation. Since hemoglobin levels in the present study were generally not significantly decreased, more data are needed to support this observation.

The primary stimulus for natriuretic peptide synthesis by myocardial cells is related to increased LV wall stress induced for example by acute myocardial infarction^43^, arterial hypertension^44^, muscle hypertrophy^45^ or increased pulmonary arterial pressure^46^. We expected that inflammation would also cause elevated BNP levels as reported earlier^47^, particularly in patients with myocarditis and dilated cardiomyopathy^48–50^. Increase of BNP has also been detected in systemic inflammation regardless of systolic function^51^. In the present study, no differences in BNP levels and LVEF were found in patients with and without myocardial inflammation, reflecting a rather compensated disease state of our study population with low rates of hydropic decompensation. Myocarditis and inflammatory cardiomyopathy are a dynamic processes; after inflammation has subsided, a reduced LVEF is due to secondary post-inflammatory fibrosis and myocardial cell death and scars^52^. As conditions with and without inflammation lead to changes of LVEF and BNP, the present study demonstrates that increase of BNP and the decrease of LVEF do not specifically reflect the presence of inflammation, but rather reflect a general loss of myocardial tissue with consecutive changes in hemodynamic conditions regardless weather inflammation can be detected or not.

## Limitation

There are certain limitations of our study which need to be mentioned: Firstly, our study was conducted as a monocentric retrospective data analysis. Therefore, it has to be emphasized that the results of this study can only serve in terms of a hypothesis generating research and the results of the present study should be verified by prospective trials. Secondly, there is no generally applicable definition of myocardial inflammation. Thus, it might be difficult to adopt the results to other endomyocardial biopsy studies, but generating more information about inflammation process was one of the main aims of our study. Finally, a potential limitation regarding EMB interpretation might be the sampling error. Regarding this point, Hauck et al. demonstrated that sampling error was prevalent 45% in Ieft and up to 37% in right EMB^53^. The authors conclude that only positive results of EMB can be considered diagnostic. However, the cited study included a very small sample of only 36 patients.

## Conclusion

Elevations of readily available markers like cardiac TnI and CRP as well as the combination of GLS and TnI may predict inflammatory cardiomyopathy and might be useful to select patients for EMB and for shorter control follow-up examinations. In the diagnostic approach to detect suspected myocardial inflammation, higher cut-off values concerning cardiac and inflammatory biomarkers may have to be applied. Smoking is associated with inflammatory cardiomyopathy in non-ischemic, non valvular HF patients and may indicate individuals at risk to develop inflammatory cardiomyopathy.

## Ethics approval

All data were obtained from individuals enrolled between 2013 and 2018 in the retrospective monocentric Mainz Endomyocardial Biopsy in Heart Failure Study (My Biopsy-HF Study, DRKS #22178), which was approved by the Ethics Committee of Rhineland Palatinate to be in accordance with the legal regulations and the declaration of Helsinki.

## Supplementary Information

Supplementary Information.

## Abbreviations

ACC: American College of Cardiology
AHA: American Heart Associaton
AUC: Area under the curve
BMI: Body Mass Index
BNP: Brain natriuretic peptide
CI: Confidence interval
CRP: C-reactive Proteine
CVRF: Cardiovascular risk factors
EMB: Endomyocardial biopsy
ESC: European Society of Cardiology
GLS: Global longitudinal strain
Hb: Haemoglobin
HF: Heart failure
HFmrEF: Heart failure with mid-range reduced ejection fraction
HFrEF: Heart failure with reduced ejection fraction
HFpEF: Heart failure with preserved ejection fraction
IQR: Interquartile range
LVEDD: Left ventricular enddiastolic diameter
LVEDP: Left ventricular enddiastolic pressure
LVEF: Left ventricular ejection fraction
NYHA: New York Heart Association
OR: Odds ratio
RNA: Ribonucleic acid
ROC: Receiver operating characteristics
TnI: Troponin I
VT: Ventricular tachycardia
