# Supplementary material for: Increased C reactive protein, cardiac troponin I and GLS are associated with myocardial inflammation in patients with non-ischemic heart failure
Source: Sci Rep. 2021 Feb 4;11:3008. doi: 10.1038/s41598-021-82592-8 (PMC7862434; doi:10.1038/s41598-021-82592-8)
Supplement: Supplementary file 1 — Supplementary Information. [file 41598_2021_82592_MOESM1_ESM.docx]

**Increased C reactive protein, cardiac troponin I and GLS are associated with myocardial inflammation in patients with non-ischemic heart failure**

Schwuchow-Thonke S^1,3^, Göbel S^1,3^, Emrich T^4^, Schmitt VH^1^, Fueting F^1^, Klank C^1^ ,Escher F^5,6^, Schultheiss HP^5^, Münzel T^1,3^, Keller K^1,2*^, Wenzel P^1,2,3*^

1. Center of Cardiology, Cardiology I, University Medical Center Mainz (Johannes Gutenberg-University Mainz), Mainz, Germany.
2. Center for Thrombosis and Hemostasis (CTH), University Medical Center Mainz (Johannes Gutenberg-University Mainz), Mainz, Germany;
3. German Center for Cardiovascular Research (DZHK), Partner Site Rhine Main, Mainz, Germany.
4. Department of Diagnostic and Interventional Radiology, University Medical Center Mainz (Johannes Gutenberg University Mainz), Mainz, Germany.
5. Institut Kardiale Diagnostik und Therapie (IKDT), Moltkestrasse 31, D-12203 Berlin, Germany
6. Departement of Internal Medicine and Cardiology, Charité – Universitätsmedizin Berlin, Campus Virchow Klinikum, Berlin, Germany
7. German Center for Cardiovascular Research (DZHK), Partner Site, Berlin, Germany

**Supplemental data**

**Figure 1:** Prognostic performance of TnI (A), BNP (B), CRP (C) and GLS (D) for prediction of inflammation (ROC curves)

*
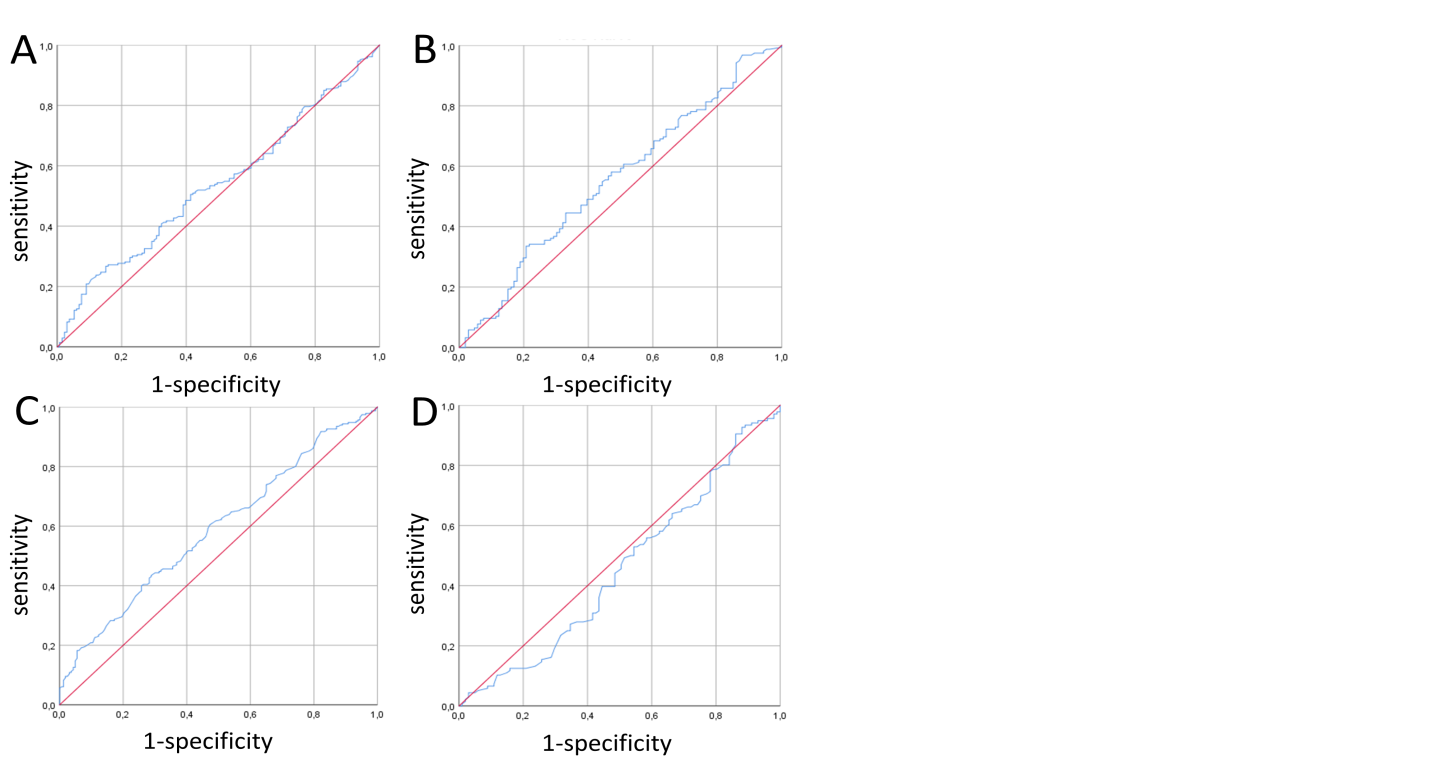
*

**Table 1:** Predictors of inflammation. OR (Odds Ratio), 95% CI (Confidence intervalls) and p-values in a crude and multivariate logistic regression analysis. GLS=global longitudinal strain in %; CRP=C-reactive protein in mg/l, TnI=Troponin I in pg/ml;

|  | **Adjustment for** | | | | | |
| --- | --- | --- | --- | --- | --- | --- |
|  | **crude** | | **+Age/Sex/Obesity** | | **+ Age/Sex/Obesity/CVRF** | |
|  | **OR [95% CI]** | **p-value** | **OR [95% CI]** | **p-value** | **OR [95% CI]** | **p-value** |
| GLS≥ -13.95%/CRP≥ 8.15 mg/l | 1.596 [0.914 – 2.788] | 0.100 | 1.601 [0.808 – 3.173] | 0.177 | 1.562 [0.781 – 3.125] | 0.207 |
| GLS≥ -13.95%/TnI≥ 136.5 pg/ml | 7.266 [2.123 – 24.863] | **0.002** | 7.655 [1.690 – 34.671] | **0.008** | 9.633 [2.027 – 45.769] | **0.004** |
| TnI≥ 136.5 pg/ml/CRP≥ 8.15 mg/ml | 2.791 [1.109 – 7.024] | **0.029** | 5.545 [1.238 – 24.837] | **0.025** | 5.761 [1.240 – 26.771] | **0.025** |

**Table 2: Prognostic performance of combined parameters to predict myocardial inflammation**. PPV, positive predictive value; NPV, negative predictive value; CI, confidence interval. GLS=global longitudinal strain in %; CRP=C-reactive protein in mg/l, TnI=Troponin I in pg/ml;

| **Parameter** | **P-value** | **Sensitivity**  **(95% CI)** | **Specificity**  **(95% CI)** | **PPV**  **(95% CI)** | **NPV**  **(95% CI)** |
| --- | --- | --- | --- | --- | --- |
| **GLS + Trop positive** | **<0.001** | 0.21 (0.15-0.29) | 0.96 (0.90-0.99) | 0.90 (0.74-0.96) | 0.46 (0.39-0.53) |
| **GLS + CRP positive** | 0.099 | 0.39 (0.31-0.47) | 0.71 (0.62-0.79) | 0.65 (0.55-0.75) | 0.46 (0.38-0.54) |
| **CRP + Trop positive** | **0.030** | 0.12 (0.08-0.17) | 0.95 (0.91-0.98) | 0.80 (0.63-0.90) | 0.41 (0.36-0.47) |
